# Hsa_circ_0000098 is a novel therapeutic target that promotes hepatocellular carcinoma development and resistance to doxorubicin

**DOI:** 10.1186/s13046-022-02482-3

**Published:** 2022-09-07

**Authors:** Yi Li, Anqi Wu, Lin Chen, Aiting Cai, Yuhao Hu, Zhou Zhou, Qianyi Qi, Yixuan Wu, Donglin Xia, Peixin Dong, Shaoqing Ju, Feng Wang

**Affiliations:** 1 Department of Laboratory Medicine, Affiliated Hospital of Nantong University, Medical School of Nantong University, Nantong, 226001 China; 2 Department of Hepatology Laboratory, Nantong Third Hospital Affiliated to Nantong University, Nantong, 226006 China; 3 School of Public Health, Nantong University, Nantong, 226019 China; 4 Department of Obstetrics and Gynecology, Hokkaido University School of Medicine, Hokkaido University, Sapporo, Japan

**Keywords:** HCC, circ_0000098, MCUR1, miR-383, P-gp, Platelet, Drug resistance

## Abstract

**Background:**

Circular RNA (circRNA) is crucial to the progression of hepatocellular cancer (HCC). In addition, Mitochondrial calcium uniporter regulatory factor 1 (MCUR1) is commonly overexpressed in HCC to increase cellular ATP levels. Due to the highly aggressive characteristics of HCC, it is essential to identify new diagnostic biomarkers and therapeutic targets that may facilitate the diagnosis of HCC and the development of effective anti-HCC treatments.

**Methods:**

A series of *in vitro* and *in vivo* experiments were undertaken to investigate the biological importance and underlying mechanisms of circ_0000098 in HCC.

**Results:**

The expression of circ_0000098 was higher in HCC tissues compared to paired adjacent tissues. According to the receiver-operating characteristic curves, circ_0000098 functioned as a potential diagnostic tumor marker in HCC. Our experiments indicated that circ_0000098 served as a key oncogenic circRNA to increase HCC cell proliferation and invasion *in vitro* and HCC progression *in vivo*. Furthermore, mechanistic investigation demonstrated that by sequestering miR-383 from the 3′-UTR of *MCUR1*, circ_0000098 positively regulated MCUR1 expression in HCC cells and finally promoted HCC progression. On the other hand, inhibiting circ_0000098 in HCC cells could diminish doxorubicin (DOX) resistance by decreasing P-glycoprotein (P-gp, MDR1) expression and intracellular ATP levels. Either downregulation of MCUR1 or overexpression of miR-383 improved DOX sensitivity in HCC cells. Subsequently, a short hairpin RNA targeting circ_0000098 (referred to as sh-1) and doxorubicin (DOX) were encapsulated into platelets (PLTs), referred to as DOX/sh-1@PLT. Activated DOX/sh-1@PLT through HCC cells resulted in the creation of platelet-derived particles that were capable of delivering the DOX/sh-1 combination into HCC cells and promoting intracellular DOX accumulation. Furthermore, our *in vivo* experiments showed that DOX/sh-1@PLT can effectively reduce P-gp expression, promote DOX accumulation, and reverse DOX resistance.

**Conclusions:**

Our results demonstrated that circ_0000098 is an oncogenic circRNA that promotes HCC development through the miR-383/MCUR1 axis and targeting circ_0000098 with DOX/sh-1@PLT may be a promising and practical therapeutic strategy for preventing DOX resistance in HCC.

**Supplementary Information:**

The online version contains supplementary material available at 10.1186/s13046-022-02482-3.

## Background

Hepatocellular carcinoma (HCC) is the second most fatal and fifth most prevalent type of cancer worldwide [1]. Despite the availability of several treatments for HCC, the overall survival rate of patients remains unsatisfactory [1]. Multiple drug resistance (MDR) diminishes chemotherapeutic effectiveness [2, 3], and the incidence rate of MDR in primary HCC ranges from 84.6% to 100%, resulting in considerable morbidity and mortality [4]. Therefore, overcoming MDR and improving drug sensitivity in cancer cells has become an important research topic in HCC treatment.

Energy supply, efflux pumps, and ATP-binding cassette (ABC) transporters are all possible MDR mechanisms that permit drug efflux, resulting in a decrease in intracellular drug concentrations and therapeutic medication effectiveness [5–8]. Mitochondria, recognized as the energy engine of a cell, are emerging as essential drivers of numerous aspects of cancer biology, such as metabolic reprogramming, acquisition of metastatic capacity, and chemotherapeutic response [9]. Calcium absorption in mitochondria is regarded to be important for cellular signaling, energy status, and survival [9]. Mitochondrial calcium uniporter regulator 1 (MCUR1) functions as a scaffold factor by binding to mitochondrial calcium uniporter (MCU) and essential MCU regulator (EMRE), and plays a crucial role in mitochondrial Ca2^+^ absorption and ATP production in cancer cells, [10, 11]. MCUR1-mediated mitochondrial calcium signaling induces the generation of reactive oxygen species (ROS) and the subsequent Notch signaling pathway, which promotes the epithelial-mesenchymal transition of HCC cells by activating ROS/Nrf2/Notch1 pathway [12]. Thus, reducing the expression of MCUR1 and inhibiting the generation of ATP in HCC might be an effective strategy for combating MDR.

Circular RNAs (circRNAs) are endogenous non-coding RNA molecules formed by back-splicing covalently-linked 3′/5′ RNA ends [13–17]. By targeting various microRNAs (miRNAs) and RNA binding proteins, circRNAs play important roles in regulating HCC cell proliferation, migration, invasion, metastasis, and chemoresistance [13–17].

Targeted drug delivery systems represent a substantial advance in cancer therapy, and biomimetic particles generated from biological sources have recently received increased attention [18, 19]. Novel innovations using platelets (PLTs) as possible carriers against cancers, in particular, are currently being intensively investigated [18, 19]. PLTs, generated by megakaryocytes, are a flexible drug delivery vehicle owing to their tiny size, superior biocompatibility, and inherent tumor-homing abilities. Recent research has demonstrated that PLTs may target tumors for accumulation by attaching to particular, cognate signal molecules on the surface of tumor cells [18, 19]. Additionally, tumor cells stimulate the aggregation of platelets and the release of their stored factors [18, 19]. Small platelet-derived microparticles enter tumor tissue more deeply, resulting in greater anti-tumor effects [20]. Because of increased CD44 on the tumor cell surface, P-selectin on the surface of PLTs may selectively bind to the surface of HCC cells [21]. Therefore, the use of PLTs to entrap MCUR1 inhibitors in HCC therapy is of great interest, as it may effectively reduce MDR in tumor tissue, resulting in a better therapeutic outcome.

In this study, we reported an oncogenic role of hsa_circ_0000098 (circ_0000098) in HCC progression by sponging miR-383 and elevating MCUR1 expression. Downregulation of circ_0000098 in HCC cells leads to a decrease in P-glycoprotein (P-gp) expression and suppression of doxorubicin (DOX) efflux. Importantly, we generated a possible circ_0000098-targeting strategy for HCC, where DOX and short hairpin RNA against circ_0000098 were entrapped inside PLTs to create platelet-coated particles (DOX/sh-1@PLT). The injection of DOX/sh-1@PLT into mouse HCC models significantly increased the sensitivity of HCC cells to DOX treatment, probably through decreasing P-gp expression and intracellular ATP generation. This study therefore identified circ_0000098 as a hopeful therapeutic target for overcoming chemoresistance in HCC and suggested a viable clinical usage for DOX/sh-1@PLT in the treatment of resistant HCC.

## Materials and methods

### Clinical samples

Fifty-eight HCC tissues and matched neighboring normal tissues were taken from HCC patients who were treated in the Nantong University’s Affiliated Hospital, from May 2013 to May 2017. All these tissues were pathologically examined and then frozen at -80 °C before being utilized. No patient had received radiation or chemotherapy before surgery. This study was approved by the Ethical Committee of Nantong University’s Affiliated Hospital, and all patients provided their informed consent.

### Cell culture

The Chinese Academy of Sciences Cell Bank (Shanghai, China) provided human HCC cell lines, including HCC-LM3, BEL-7404, and SK-Hep1, as well as human normal liver cell line LO2. HCC-LM3, BEL-7404, and SK-Hep1 cells were cultured in Dulbecco's modified Eagle's medium (DMEM, Corning, USA) supplemented with 10% fetal bovine serum (FBS, Lonsera, USA) at 37 °C and 5% CO_2_ in a humidified atmosphere. Under the same culture conditions, LO2 cells were cultured in RPMI-1640 medium (Corning, NY, USA).

### RNA extraction and quantitative real-time PCR (RT-qPCR)

TRIzol® (Thermo Fisher Scientific, USA) was used to extract total RNA from tissues and cells at 4 °C, followed by quantitative real-time PCR (RT-qPCR). RNA was reverse-transcribed using the Reverse Transcription Kit® (ThermoFisher Scientific, USA) to create cDNA, which was then amplified using the LightCycler 480 SYBR Green I Master Mix (Roche, Germany). The expression of the amplification products was calculated using the delta-delta comparative (2^−ΔΔCT^) approach. All of the experiments were done at least three times. Bulge-loopTM miRNA RT-qPCR primer sets against miR-383 and U6 were designed by RiboBio (Guangzhou, China). Table 1 lists the remaining the primers that were used in this study. GENE (Shanghai, China) provided the primers for circ_0000098, whereas the primers for *MCUR1* and 18S RNA were purchased from Sangon Biotech (Shanghai, China).

**Table 1:** Sequences of primers

Gene name	Primer sequences
hsa_circ_0000098	F: 5'- CAGGACCCAGCAGACAGATT-3' R: 5'-ATCAAGCCTAAGCTTACAAC-3'
SLC30A7	F: 5'- AGAG CATTAGCCCCTCCAGA-3' R: 5'-GGCCAGAGCCATGAGAATGT-3'
MCUR1	F: 5'- CAGGAAACTCTACTTCGACACT-3' R: 5'-GACCAATGCAGACACAATGATT -3'
18sRNA	F: 5'-GTAACCCGTTGAACCCCATT-3' R: 5'-CCATCCAATCGG TAGTAGCG-3'

### Construction and transfection of vectors

GENE (Shanghai, China) created the shRNA vectors targeting circ_0000098 and MCUR1, as well as a circ_0000098 overexpression vector (named OE) or control vector (Table 2).　RiboBio (Guangzhou, China) designed and manufactured the miR-383 mimics or control mimics. HCC cells were equally distributed onto the plates and cultured to a density of 70–80%, after which the media was changed to a basic medium lacking FBS. Using Lipofectamine 3000® (ThermoFisher Scientific, USA), plasmids were transfected into cells. The adenovirus utilized in this study was created and generated by GENE (Shanghai, China).

**Table 2:** Sequences of plasmids

Plasmid name	Plasmid sequence
sh-hsa_circ_0000098	sh-1: 5’-GCCATTCTTATAGTTGTAAGCTTAG-3’
	sh-2: 5’-TGTAAGCTTAGGCTTGATT-3’
sh-MCUR1	5’- GCTGGAATT GAGAACAGAAAT-3’

### Assessment of cell proliferation

The cellular proliferative rates were determined using a Cell Counting Kit-8® (CCK-8, MCE, China). 100 µl of cell suspension with a density of 3 × 10^4^/ml was added to each well of a 96-well plate, and 10 µl of CCK-8 reagent was added at the indicated times. The absorbance at 450 nm was measured to evaluate cell proliferation after 2 h of incubation. A colony formation assay was used to measure the capacity of cells to proliferate. 600 HCC cells were seeded into six-well plates. After 14 days of culture, the cells were fixed and stained, and the total colonies formed were counted.

### Cell invasion and migration assays

Cell invasion tests were carried out in 8-µm Transwell chambers. HCC cells were resuspended in FBS-free basal media and inoculated on the upper chamber pre-plated with 100 µl of diluted Matrigel (Corning, USA). The bottom chamber was filled with an FBS-enriched complete medium. After 48 h of culture, the chamber was fixed with paraformaldehyde and stained with 0.5% crystal violet. The crystal violet was then rinsed away, and the cells were counted using a microscope. The migration experiment was similar to the invasion experiment, with the exception that Matrigel was not utilized to cover the upper chamber. After being fixed and dyed, the migrated cells were viewed under a microscope.

### Flow cytometry assays

HCC cells were rinsed twice in prechilled PBS before being fixed overnight in 70% ethanol at 4 °C, followed by 50 µl of RNase A (Solarbio, China) to resuspend these cells. The cells were then stained with 200 µl of propidium iodide (PI) staining solution (Solarbio, China) at 37 °C for 60 min and 4 °C for 30 min in the dark. Cell cycle analysis was performed using a BD FACSCanto™ II flow cytometer (BD, USA). Furthermore, the apoptotic rate of cells was determined using a BD Pharmingen™ FITC Annexin V apoptosis detection kit® (BD, USA). A FACSCalibur flow cytometer (BD, USA) was used for apoptosis analysis after the cells were processed according to manufacturer instructions.

### Western blot assays

To collect and lyse HCC cells, radioimmunoprecipitation assay buffer (RIPA, Solarbio, China) was used. To decrease protease activity, phenylmethylsulfonyl fluoride (Solarbio, China) was added throughout the lysis process. After measuring total cell protein concentrations using a NanoDrop One ultraviolet spectrophotometer (Thermo Scientific), a protein loading buffer (Beyotime Institute of Biotechnology, China) was added, and samples were heated to denature proteins. After that, the protein samples were isolated using a 10% PAGE Gel Fast Preparation Kit (EpiZyme, China) and transferred on 0.45-μm polyvinylidene fluoride membranes (PVDF, Millipore, Germany). The PVDF membranes were then blocked at room temperature for 120 min. PVDF membranes were treated with primary antibodies overnight at 4 °C after being diluted at 1:1000. Then, membranes were incubated with appropriate secondary antibodies (Abcam, USA) for 1 h, and the bands were detected using enhanced chemiluminescence reagents (New Cell & Molecular Biotech, China) and quantified using ImageJ software (NIH Image, Bethesda, MD). The following primary antibodies were used in this study: MCUR1 (Cell Signaling Technology, USA), GAPDH (Absin, China), and P-gp, GP IIb and GP VI (Abcam, UK).

### RNA Immunoprecipitation assays(RIP)

First, the collected cell samples were extracted and pretreated according to the method provided by the manufacturer (Geneseed, China). The antibody was then attached to the pretreated magnetic beads and the antigen was captured. Finally, the extracted RNA was stored at -80 °C for later use.

### Mouse xenograft HCC models

Four-week-old male BALB/c nude mice were obtained from Nantong University's Laboratory Animal Centre (China), and used under protocols approved by the aforementioned center for in vivo antitumor study. HCC-LM3 cells transfected with control shRNA (sh-NC) and shRNA against circ_0000098 (sh-1) were given subcutaneously to mice in the axillary system. Every day, the tumor volume of naked mice was inspected and recorded. Mice were sacrificed 7, 14, and 21 days after being exposed to isoflurane, and tumors were removed to weigh and calculate their volume. All efforts have been made to minimize animal suffering. The formula for calculating tumor volume (mm^3^) was [tumor long diameter × tumor short diameter^2^]/2. All animal experiments and procedures involving animal euthanasia were carried out in accordance with the relevant laws and institutional guidelines of the Department of Medicine of Nantong University and were approved by the Animal Ethics Committee of Nantong University.

### PLTs extraction

Whole blood samples from healthy subjects were centrifuged (200 g, 10 min) before upper-serum collection. To inhibit PLT aggregation, 0.5 mg of PGE1 was given to the serum. To produce a PLT pellet, the serum was centrifuged (1800 g, 20 min).

### Preparation of DOX/sh-1@PLT

First, 100 μl of DOX solution and 30 μg of sh-1 were added to 250 μl of PLT solution, and the resulting combination was sonicated for 6 min at 50 kHz and 100 W. PBS was then added, and the resulting mixture was centrifuged (1800 g, 20 min) to separate unbound free DOX and sh-1 from the supernatant. Finally, 250 μl of PBS was added to the precipitate, yielding the product DOX/sh-1@PLT [18, 19].

### Establishment of orthotopic HCC mice models

First, BALB/c nude mice were anesthetized with chloral hydrate, the abdomen was opened, 1 × 10^6^ HCC-LM3 cells were injected into the proper portion of the liver, and the abdomen was closed. Then, beginning on day 0 or day 14, normal saline, DOX, DOX/sh-NC@PLT, and DOX/sh-1@PLT were administered every two days into the tail veins of nude mice. Finally, experimental data was gathered on days 9, 18, 28, or 56. All experimental procedures involving animal use and euthanasia were carried out in accordance with the relevant laws and institutional guidelines of the Department of Medicine of Nantong University and were approved by the Animal Ethics Committee of Nantong University.

### ATP content detection

The cells were collected in a centrifuge tube for ATP content detection, and the ATP levels were measured according to the manufacturer-supplied methodology (Solarbio, China).

### 3-D tumor ball assay

The digested HCC cells were added to a round-bottom 96-well plate at a density of 3 × 10^6^/ml, and 3-D spherical cell clusters were formed after culturing for 72 h. Subsequently, 100 μl DOX and DOX/sh-1@PLT were added to the well plate and cultured for 12 h, and the DOX fluorescence status of each layer (I, II, III, and IV) of the tumor ball was observed using a laser confocal microscope.

### Fluorescence in situ hybridization

The cells were evenly seeded in a 24-well plate, and when the cell confluence reached about 70%, the cells were fixed and the cell permeability was increased. Cells were then assayed using probes (Ribobio) containing circ_0000098 or GAPDH or U6 according to the RiboTM Fluorescent In Situ Hybridization Kit instructions. Finally, the nuclei were stained with DAPI and the cells were examined by fluorescence microscopy.

### Statistical analysis

Statistical analysis was performed using SPSS22 (IBM, USA). The results are shown as the mean ± standard deviation. Regarding the comparison between the two groups, a two-tailed Student's *t*-test was used, and a one-way ANOVA was utilized to compare the data amongst the different groups. *P* < 0.05 was considered statistically significant.

## Results

### Circ_0000098 is overexpressed in HCC

**Fig. 1:**
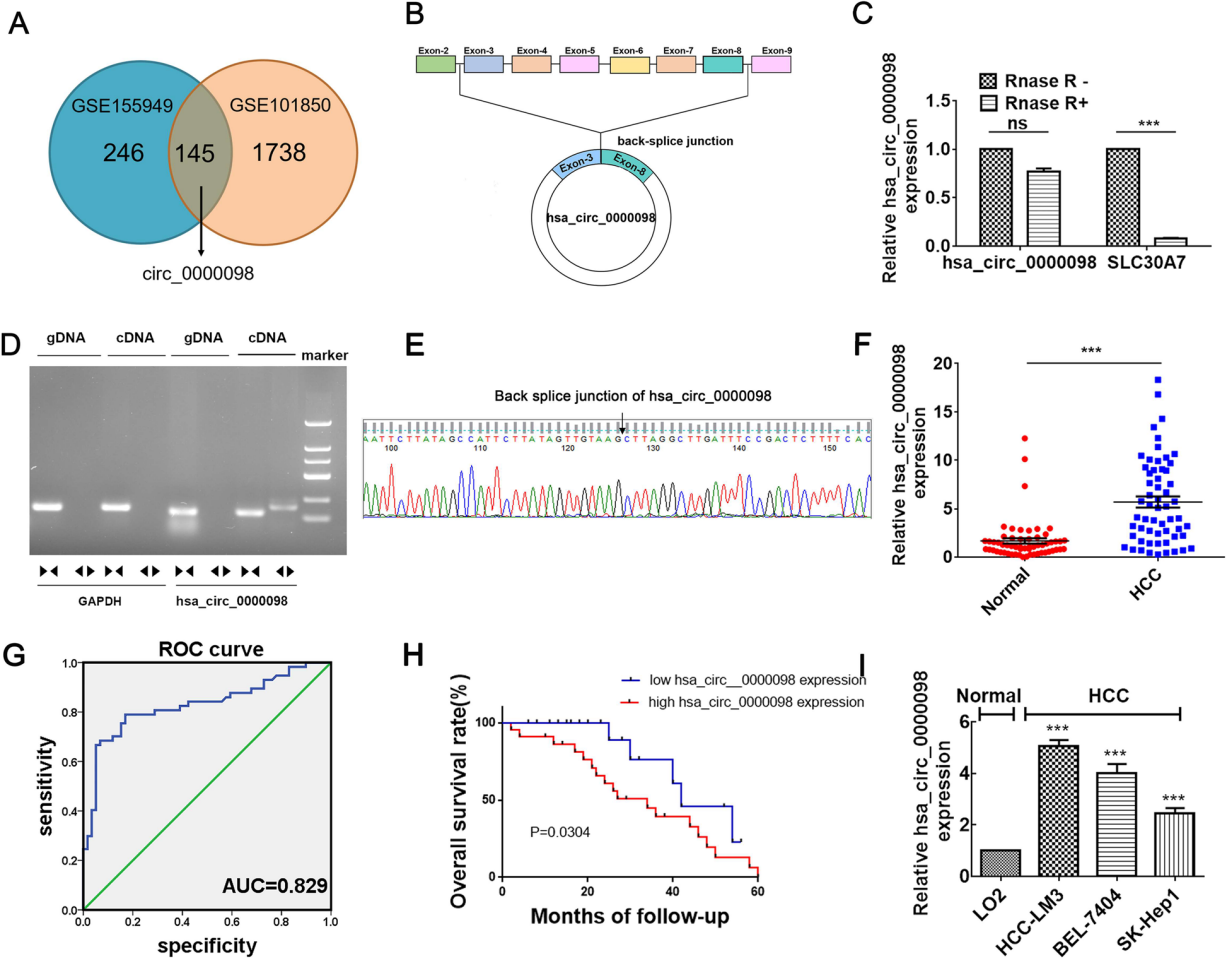
Circ_0000098 is highly expressed in HCC. **A** Venn diagrams from analysis of GSE155949 and GSE101850 datasets. **B** Illustration of circ_0000098 formation from *SLC30A7* gene. **C** The stability of circ_0000098 and *SLC30A7* mRNA was evaluated following RNase R treatment using RT-qPCR assays. **D** Assay of PCR-amplified products of circ_0000098 on an agarose gel electrophoresis. Divergent primers were capable of amplifying circ_0000098 inside cDNA but not genomic DNA (gDNA). *GAPDH* served as a linear reference, while convergent primers amplified both circ_0000098 and linear gDNA. **E** The back-splicing junction of circ_0000098 was validated using Sanger sequencing. **F** Comparison of circ_0000098 levels in HCC and adjacent normal tissues. **G** ROC curve analysis of circ_0000098 for distinguishing HCC from healthy tissues. **H** Kaplan–Meier curve analysis of the prognosis of HCC patients with high or low circ_0000098 expression. **I** RT-qPCR was used to detect circ_0000098 expression in HCC cells (HCC-LM3, BEL-7404, and SK-Hep1) and a normal cell line LO2. NS: not significant. ***P < 0.001

First, to reveal differentially expressed circRNAs,we selected GSE155949 dataset and GSE101850 dataset for evaluation. We randomly selected 15 pairs of HCC and adjacent normal tissue samples from the GSE155949 dataset and 3 pairs of HCC cells and drug-resistant HCC cells from the GSE101850 dataset for analysis.In the GSE155949 dataset, 392 molecules were up-regulated and 218 were down-regulated. Among the up-regulated molecules, 145 molecules intersected with 1,738 up-regulated molecules in the GSE101850 dataset. Among them, circ_0000098 was significantly up-regulated, and we selected it for further study (Fig. 1A). The circular structure of circ_0000098 is generated from exons 3–8 of its host gene, *SLC30A7*, according to the circBank database (www.circBank.cn) (Fig. 1B). RNase R was used to validate the circular characteristics of circ_0000098 in HCC-LM3 cells. In contrast to the linear *SLC30A7* mRNA, circ_0000098 was resistant to RNase R treatment (Fig. 1C). Furthermore, RT-qPCR assays using divergent primers confirmed that circ_0000098 was stably expressed in HCC tissues (Fig. 1D). Sanger sequencing of the RT-qPCR products amplified by divergent primers verified the back-splicing junction sequence (Fig. 1E). The RT-qPCR assay was used to identify the expression of circ_0000098 in 58 paired HCC tissue and adjacent normal tissues. The findings showed that the levels of circ_0000098 were significantly elevated in HCC tissues compared to normal tissues (Fig. 1F). Receiver operating characteristic (ROC) curves were utilized to determine whether circ_0000098 might be a diagnostic tumor marker for HCC. The area under the ROC curve was 0.829, showing its potential diagnostic value for HCC (Fig. 1G). Meanwhile, our investigation showed that HCC patients who showed high levels of circ_0000098 expression had shorter survival rates than those with low levels of circ_0000098 (Fig. 1H). Furthermore, circ_0000098 was found to be overexpressed in HCC cell lines (HCC-LM3, BEL-7404, and SK-Hep1) compared with normal cells (Fig. 1I). These results implied that elevated circ_0000098 played a critical role in the development of HCC.

### Circ_0000098 stimulates HCC development *in vitro*

**Fig. 2:**
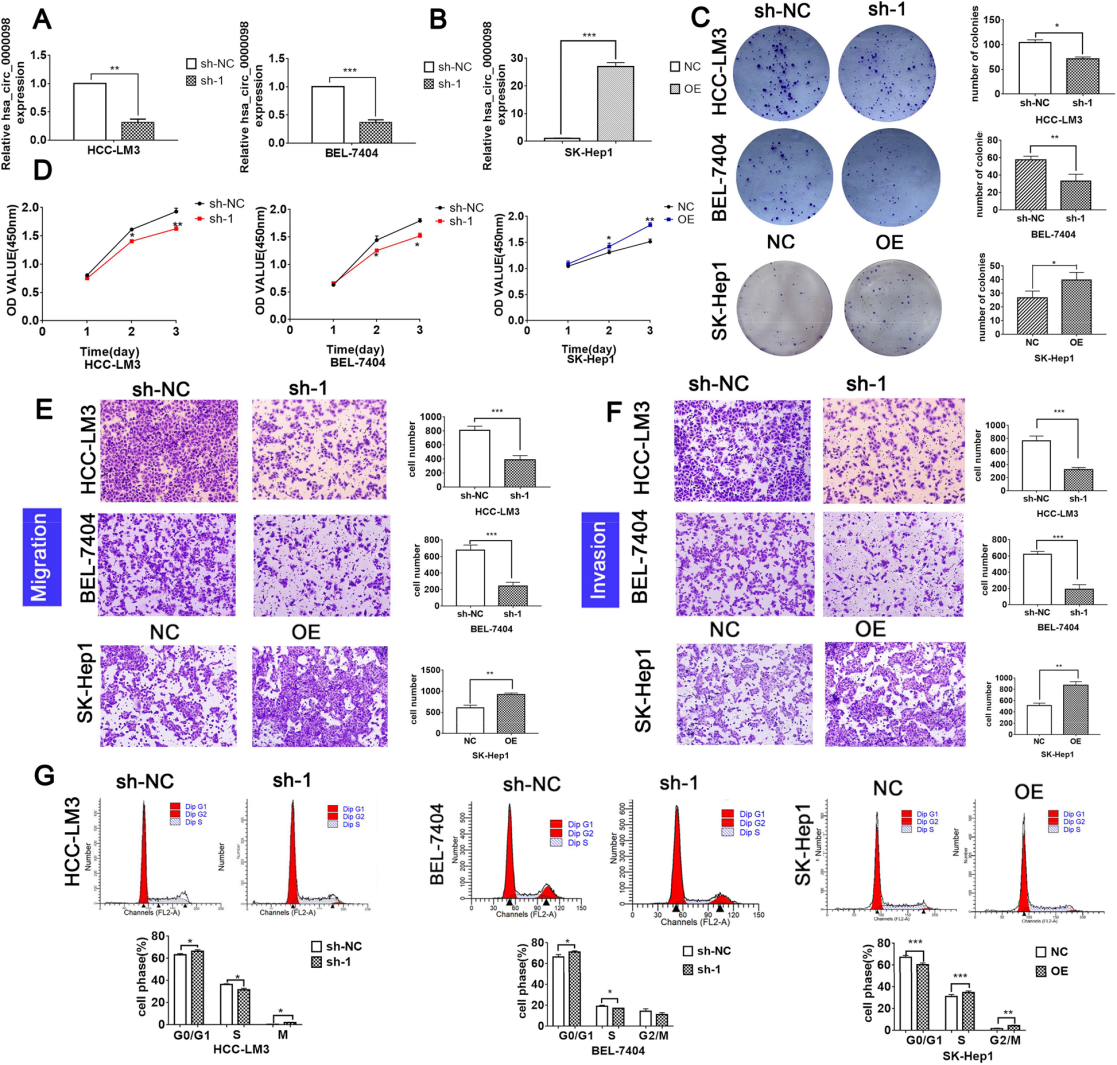
circ_0000098 promotes HCC cell growth and invasiveness *in vitro.* **A**, **B** RT-qPCR detection of circ_0000098 expression in HCC-LM3 and BEL-7404 cells transfected with the shRNA vector targeting circ_0000098 (sh-1) or control shRNA (sh-NC) (**A**), and in SK-Hep1 cells transfected with the circ_0000098 overexpression vector (OE) or control vector (NC) (**B**). **C-G** Clone formation assays (**C**), CCK-8 assays (**D**), migration assays (**E**), invasion assays (**F**), and flow cytometry analysis (**G**) were carried out in HCC cells with circ_0000098 knockdown or overexpression. *P < 0.05, **P < 0.01, ***P < 0.001

To examine the biological role of circ_0000098 in HCC, the shRNA vector targeting circ_0000098 (sh-1) and a circ_0000098 overexpression vector (OE) were created. Approximately 70% interference effectiveness was observed after sh-1 transfection into HCC-LM3 and BEL-7404 cells (Fig. 2A), and a 25-fold increase in circ_0000098 expression was found in SK-Hep1 cells transfected with OE (Fig. 2B). These results showed that the two above vectors were successfully generated. Our clone formation and CCK-8 assays suggested that sh-1 significantly decreased the number of clones and inhibited HCC cell proliferation, while OE significantly increased the number of clones and promoted HCC cell proliferation (Fig. 2C; D). In addition, transwell migration and invasion experiments demonstrated that sh-1 efficiently inhibited HCC cell migration and invasion, while OE had the reverse effects (Fig. 2E; F). Moreover, flow cytometry experiments demonstrated that sh-1 could significantly induce cell-cycle arrest and increase the number of apoptotic HCC cells (Fig. 2G; Fig. S1A-C). Conversely, overexpression of circ_0000098 accelerated cell cycle progression and inhibited apoptosis in HCC cells (Fig. 2G; Fig. S1A-C). The above findings suggested that circ_0000098 acts as an oncogene to promote HCC development *in vitro*.

### Knockdown of circ_0000098 inhibits HCC tumor growth *in vivo*

In addition, xenograft HCC models were constructed in null mice, and we found that compared with the sh-NC group, the tumor dimensions and weight of the sh-1 group were much smaller and the circ_0000098 content decreased (Fig. S2A-E). Simultaneously, hematoxylin–eosin (HE) staining and immunohistochemical determination of Ki67 were performed on resected tumor tissues, and the results indicated more necrotic tumor cells in the sh-1 group (Fig. S2F), demonstrating that inhibition of circ_0000098 could repress HCC cell growth capability in vivo.

### Circ_0000098 increases MCUR1 expression via sponging miR-383

**Fig. 3:**
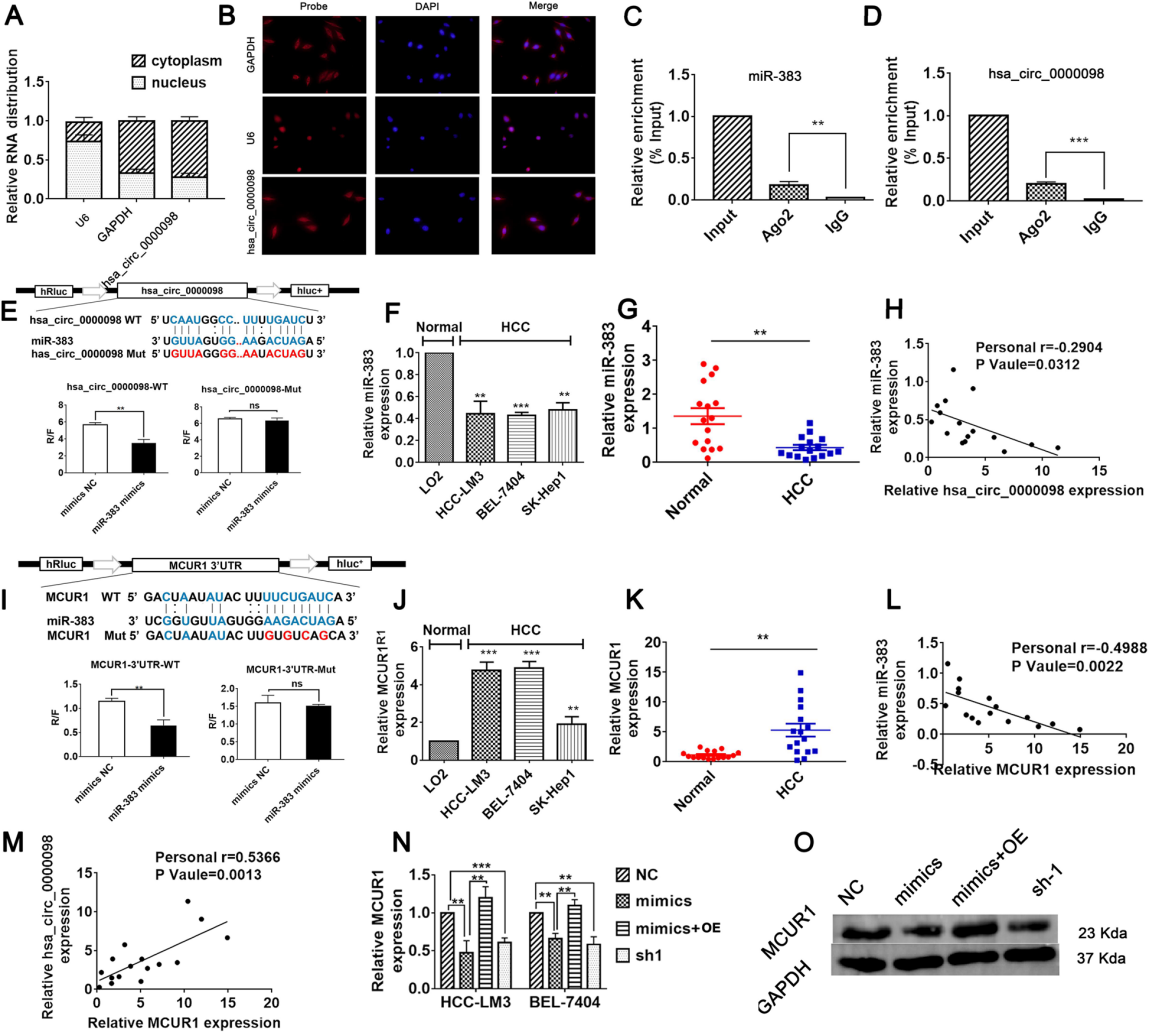
Circ_0000098 regulates MCUR1 expression by sponging miR-383. **A** The cytoplasmic and nuclear levels of circ_0000098 in HCC-LM3 cells were examined using RT-qPCR assays. *GAPDH* and U6 were utilized as cytoplasmic and nuclear controls, respectively. **B** FISH assay examined the localization of circ_0000098 in HCC-LM3 cells. GAPDH and U6 were utilized as cytoplasmic and nuclear controls, respectively. **C**, **D** The Ago2 RIP assay was used to evaluate miR-383 (**C**) and circ_0000098 (**D**) expression in HCC-LM3 cells. **E** Bioinformatics prediction of the binding sites between circ_0000098 and miR-383. Wild-type (WT) and mutant (Mut) binding sequences for circ_0000098 were cloned into a luciferase reporter. Relative luciferase activity (R/F) was analyzed for HCC-LM3 cells co-transfected with miR-383 mimics, control mimics (mimics NC), along with WT or Mut luciferase reporter vectors. **F** Detection of miR-383 expression in HCC cell lines and LO2 cells by RT-qPCR. **G** Examination of miR-383 expression within HCC and adjacent normal tissues by RT-qPCR. **H** Correlation analyses of miR-383 and circ_0000098 expression in HCC tissues. **I** Bioinformatics predictions for binding sites between miR-383 and *MCUR1* 3′-UTR. The WT and Mut binding sequences of *MCUR1* 3′-UTR were cloned into the luciferase reporter. R/F was analyzed in HCC-LM3 cells co-transfected with miR-383 mimics or mimics NC, together with WT or Mut luciferase reporter vectors. **J** Detection of *MCUR1* mRNA expression in HCC cells and LO2 cells using RT-qPCR assays. **K** Detection of *MCUR1* mRNA expression in HCC and adjacent normal tissues by RT-qPCR. **L**, **M** Correlation analyses of miR-383 and *MCUR1* mRNA expression (**L**) and circ_0000098 and *MCUR1* mRNA levels (**M**) in HCC tissues. **N** Detection of *MCUR1* mRNA expression in HCC cells transfected as indicated using RT-qPCR assays. **O** Western blotting analysis of MCUR1 expression in the indicated cells. **P < 0.01, ***P < 0.001, NS: not significant

To investigate the molecular mechanisms behind circ_0000098 in HCC, its localization in HCC cells was examined. Nuclear and cytoplasmic separation studies and FISH assays determined that the majority of circ_0000098 was localized in the cytoplasm (Fig. 3A-B). CircRNAs in the cytoplasm may serve as a sponge for miRNA in cells [13–17]. Therefore, we hypothesized that circ_0000098 interacts with miRNAs in HCC cells. Using microRNA target scanning algorithm software, we found that circ_0000098 contains the binding site for miR-383. RNA immunoprecipitation experiments demonstrated that both miR-383 and circ_0000098 can bind to Ago2, suggesting that they may interact with each other (Fig. 3C-D). Dual-luciferase analysis revealed a direct interaction site between miR-383 and circ_0000098 (Fig. 3E). In addition, by using the TCGA and KM mapper databases, we found that miR-383 expression levels were lower in HCC tissues, and lower levels of miR-383 were associated with poor patient prognosis (Fig. S3A-B). Subsequently, RT-qPCR experiments showed that the expression of miR-383 was significantly downregulated in HCC cells and HCC tissues (Fig. 3F-G). At the same time, the correlation study showed that the expression of miR-383 in HCC tissues was negatively correlated with the expression of circ_0000098 (*r* = -0.2904, *P* = 0.0312) (Fig. 3H).

In addition, our prediction identified a putative miR-383 binding site in the 3'-untranslated region (3'-UTR) of *MCUR1* mRNA, to test whether miR-383 binds to *MCUR1* mRNA, we transfected miR-383 mimics or control mimics to HCC cells (Fig. S3C-D). The RT-qPCR assays confirmed the upregulation of miR-383 in HCC cells transfected with miR-383 mimic (Fig. S3C-D). Then, we predicted the binding site of miR-383 to *MCUR1* by microRNA target scanning algorithm software, and confirmed that miR-383 directly interacts with *MCUR1* by dual-luciferase analysis (Fig. 3I).Database analysis suggested that MCUR1 levels were elevated in HCC tissues and higher levels of MCUR1 expression were associated with poor prognosis (Fig. S3E-F).

To systematically understand the biological functions and signaling pathways associated with miR-383, its target genes were identified using the ENCORI database (https://starbase.sysu.edu.cn/index.php). As a result, a total of 1592 genes were identified. To understand the molecular interaction networks that were controlled by miR-383, these target genes were investigated using the ENCORI algorithm for the KEGG pathway and GO annotation analysis. The KEGG pathway analysis showed that potential target genes were enriched in 13 pathways. Among the most enriched categories were pathways in cancer, regulation of actin cytoskeleton, renal cell carcinoma, p53 signaling, and focal adhesion pathways (Fig. S4A). Analysis of GO annotations indicated that miR-383 targets are strongly associated with adherens junction formation, cell cycle, and cell death (Fig. S4B). These findings suggest that miR-383, a circ_0000098-interacting miRNA, may modulate multiple biological signaling pathways in HCC (especially cancer metastasis-related pathways).

Subsequently, by RT-qPCR, we found that *MCUR1* expression was elevated in HCC cells and HCC tissues (Fig. 3J-K). As expected, the expression of miR-383 was conversely correlated with the expression of *MCUR1* in HCC tissues (*r* = -0.4988, *P* = 0.0022) (Fig. 3L). Of note, circ_0000098 expression had a positive correlation with *MCUR1* expression (Fig. 3M). MCUR1 mRNA and protein levels were significantly decreased in HCC cells transfected with miR-383 mimics or sh-1 (Fig. 3N and O). When circ_0000098 expression was overexpressed in HCC cells, MCUR1 levels were significantly recovered (Fig. 3N and O). These findings demonstrated that circ_0000098 binds miR-383, releasing its competitively repressive effects and increasing MCUR1 expression.

### Circ_0000098 promotes HCC development and P-gp expression via the miR-383/MCUR1 axis

**Fig. 4:**
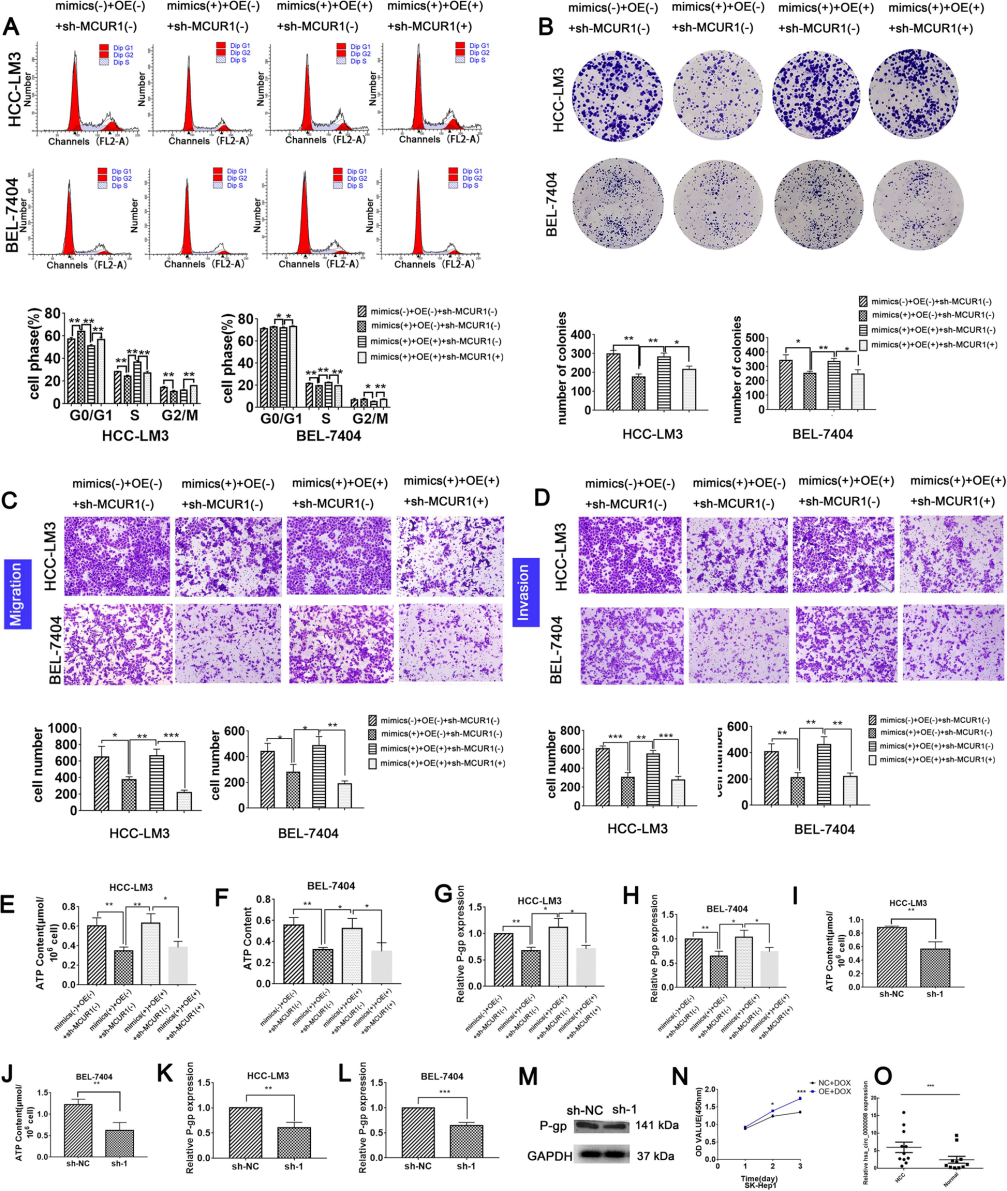
Circ_0000098 promotes HCC development and P-gp expression via the miR-383/MCUR1 axis. **A-D** Flow cytometry analysis (**A**), clone formation assays (**B**), migration assays (**C**), and invasion assays (**D**) were conducted to detect cell cycle arrest in the indicated cells. **E–F** Detection of ATP levels in HCC-LM3 (**E**) and BEL-7404 (**F**) cells transfected as indicated. **G-H** RT-qPCR assays were used to detect P-gp expression in HCC-LM3 (**G**) and BEL-7404 (**H**) cells transfected as indicated. **I-J** Detection of ATP levels in HCC-LM3 (**I**) and BEL-7404 (**J**) cells transfected as indicated. **K-L** RT-qPCR assays were used to detect P-gp expression in HCC-LM3 (**K**) and BEL-7404 (**L**) cells transfected as indicated. **M** Detection of P-gp protein expression in sh-1- and sh-NC-transfected HCC-LM3 cells using western blotting analysis (**N**) The effect of circ_0000098 on extracellular DOX resistance in HCC was detected by CCK-8 assay (**O**) The expression of circ_0000098 in DOX-resistant patients. *P < 0.05, **P < 0.01, ***P < 0.001

The shRNA expression vector targeting MCUR1 (sh-MCUR1) was successfully created (Fig. S3G-H). Cell cycle progression, cell proliferation, colony formation, migration, and cell invasion were significantly reduced in miR-383 mimic-treated HCC cells as compared to the control group (Fig. 4A-D; S5A-B). However, these effects of miR-383 were partially rescued by OE (Fig. 4A-D; S5A-B). As predicted, sh-MCUR1 significantly suppressed HCC growth, migration, and invasion (Fig. 4A-D; S5A-B). To verify the association between circ_0000098, miR-383, and MCUR1 *in vivo*, the RT-qPCR assays were performed to examine their levels in xenograft HCC tissues. The results showed that the knockdown of circ_0000098 expression resulted in the downregulation of *MCUR1* and an increase in miR-383 expression (Fig. S6A-C). Overall, our results demonstrated that circ_0000098 may enhance the malignant development of HCC through the miR-383/MCUR1 axis.

In addition, we hypothesized that circ_0000098 might influence anti-cancer drug efflux through the miR-383/MCUR1 axis. The results from an ATP content measurement and RT-qPCR assays indicated that the ATP levels, as well as the levels of the ATP-dependent drug efflux protein P-gp were reduced in HCC cells transfected with miR-383 mimics (Fig. 4E-H). However, the upregulation of circ_0000098 increased ATP levels and enhanced the expression of *P-gp* in HCC cells (Fig. 4E-H). By analyzing the KM plotter database, we found that high expression of *ABCB1* gene (P-gp) was associated with poor survival in HCC patients (Fig. S6D). Moreover, downregulation of MCUR1 or circ_0000098 in HCC cells was able to repress ATP production and the expression of P-gp (Fig. 4I-M). Subsequently, further studies showed that in DOX-treated HCC cell lines, overexpression of circ_0000098 could increase the DOX resistance of HCC cells *in vitro* (Fig. 4N). And the content of hsa_circ_0000098 was higher in DOX-resistant patients (Fig. 4O).The findings indicated that dysregulation of the circ_0000098/miR-383/MCUR1 signaling pathway leads to P-gp overexpression and elevated ATP levels, both of which may promote anti-cancer drug efflux and chemoresistance in HCC.

### Preparation and characterization of DOX/sh-1@PLT

**Fig. 5:**
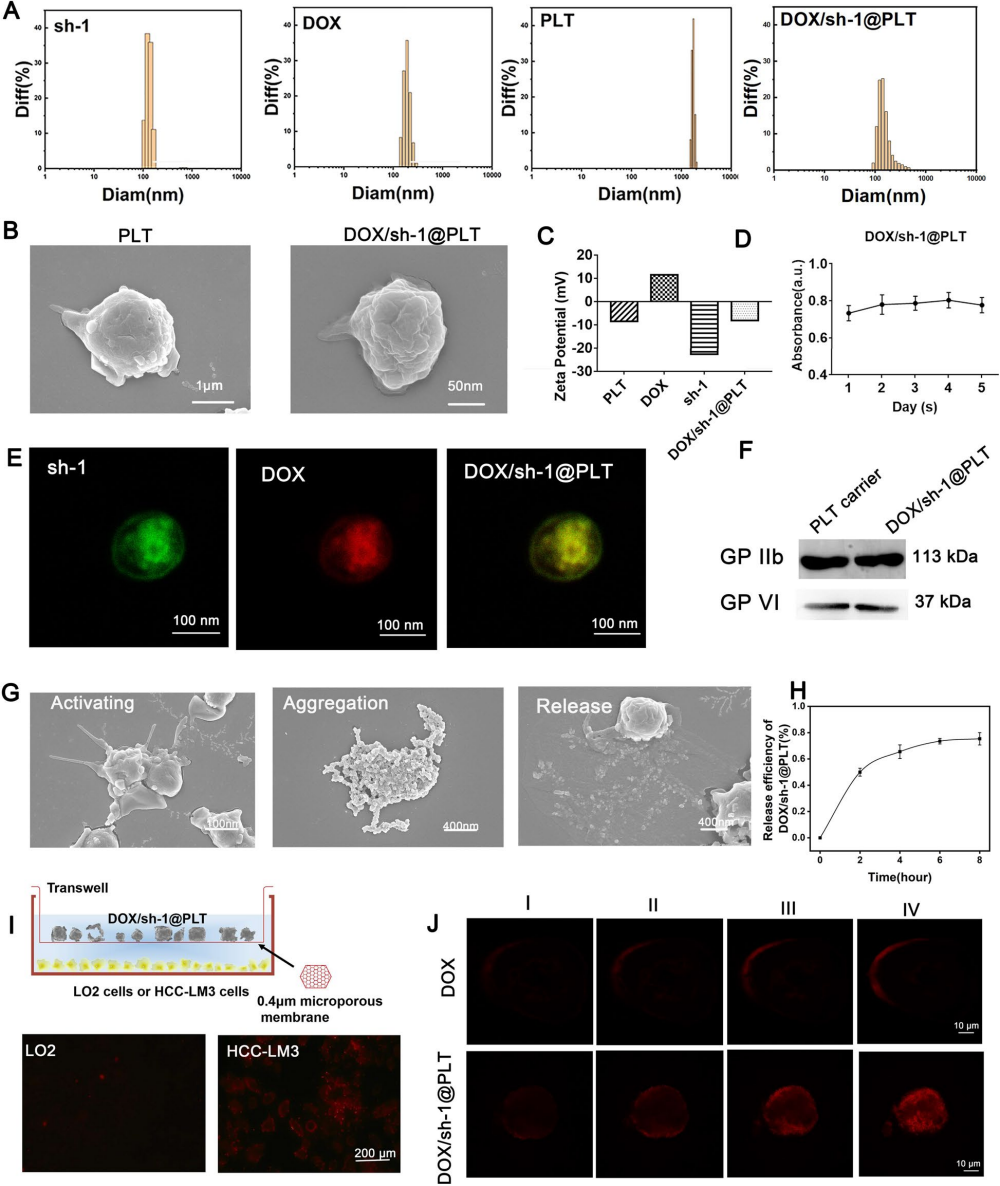
Preparation and characterization of DOX/sh-1@PLT. **A** and **C** Particle size analysis and zeta potential analysis technologies were used to detect sh-1, DOX, PLTs, and DOX/sh-1@PLTs. **B** Detection of PLT and DOX/sh-1@PLT by SEM. **D** Detection of the stability of DOX/sh-1@PLT by UV spectrophotometry. **E**–**F** Characterization of DOX/sh-1@PLT by laser confocal microscopy (**E**) and western blot assay (**F**). **G** Detection of the excitation process of DOX/sh-1@PLT by SEM. **H** Detection of the release rate of DOX/sh-1@PLT by UV spectrophotometry. **I-J** Detection of the ability of DOX/sh-1@PLT to target HCC cells by transwell chamber assay (**I**) and the 3D tumor ball assay (**J**)

Recent studies have shown that during tumor progression, activated PLT containing P-selectin can selectively adhere to tumor cells and thus better penetrate the tumor [20]. At the same time, due to its small size and good biocompatibility, PLT has attracted more attention as an innovative drug delivery carrier [18, 19]. To evaluate the impact of sh-1 in the treatment of HCC by blocking drug efflux, PLT was used as a carrier to encapsulate the anti-cancer medication DOX with the sh-1 plasmid vector to form DOX/sh-1@PLT [22, 23]. Simultaneously, DOX/sh-NC@PLT was created as a control. According to particle size analysis technology, the particle size of DOX/sh-1@PLT was around 220 nm, indicating that it may pass through the tumor blood vessel wall and infiltrate the tumor tissue as drug-carrying nanoparticles (Fig. 5A). We observed the DOX/sh-1@PLT drug morphologies using scanning electron microscopy (SEM) and discovered that the surface of the PLTs became rough after DOX and sh-1 encapsulation, resulting in a distinct morphology (Fig. 5B). According to zeta potential analysis methods, the DOX/sh-1@PLT potential was -9.93 mV, which was consistent with the electronegativity required by the human body (Fig. 5C). UV spectrophotometry was employed to demonstrate the high stability of DOX/sh-1@PLT (Fig. 5D). A laser confocal microscope was then used to photograph DOX/sh-1@PLT. Under excitation light, red fluorescence (DOX fluorescence) and green fluorescence (sh-1 fluorescence) were observed in DOX/sh-1@PLT (Fig. 5E), demonstrating that both sh-1 and DOX were successfully encapsulated in PLT. This finding was further validated using western blot experiments (Fig. 5F). Using scanning electron microscopy (Fig. 5G) and particle size analysis technology (Fig. S7), we discovered that when DOX/sh-1@PLT was activated *in vitro*, visible tentacles with a particle size of about 400 nm emerged on the periphery. The engineered PLTs then clumped together to form clusters, eventually increasing the particle size of the composite. Finally, DOX and sh-1 were released gradually, and the particle size of DOX/sh-1@PLT dropped steadily (Fig. 5G). UV spectrophotometry revealed that the release rate of DOX/sh-1@PLT was approximately 75% (Fig. 5H). Following the successful creation of DOX/sh-1@PLT, we investigated its influence on the tumorigenesis of HCC. To this end, transwell chamber assays and 3D tumor ball assays were utilized to investigate the impact of DOX/sh-1@PLT on HCC cells, and our results suggested that DOX/sh-1@PLT accumulated more in HCC cells but less in normal cells (Fig. 5I), and effectively targeted HCC cells to release DOX while enhancing the intracellular accumulation of DOX (Fig. 5J).

### DOX/sh-1@PLT inhibits HCC development and DOX resistance in vivo

**Fig. 6:**
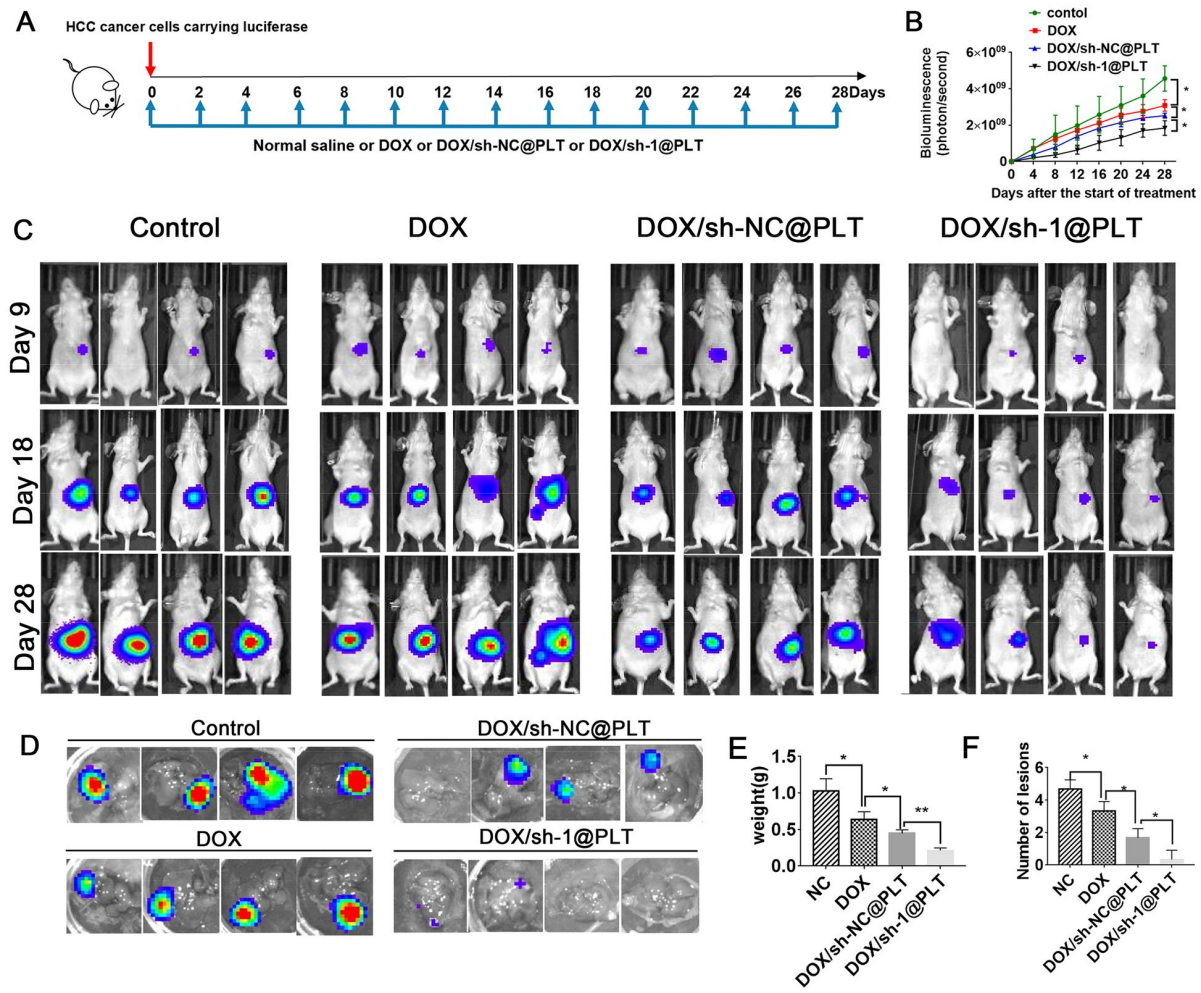
DOX/sh-1@PLT inhibits HCC development and DOX resistance *in vivo*. **A** Schematic illustration of the treatment regimen. **B-C** Fluorescence intensity alterations (**B**) and representative images (**C**) in HCC model mice were detected. **D** Detection of fluorescence changes of intensity in livers of HCC-bearing mice. **E** Detection of tumor weight. **F** Detection of the number of tumor lesions. *P < 0.05, **P < 0.01

To investigate the effect of DOX/sh-1@PLT on HCC formation *in vivo*, we established mouse HCC models. Physiological saline, DOX/sh-NC@PLT, and DOX/sh-1@PLT were injected every two days for 28 days (Fig. 6A). The fluorescence intensity of HCC cells in nude mice was measured using *in vivo* imaging technologies. Mice treated with DOX showed a weaker fluorescent signal when compared to the control group, and the fluorescent signal was further diminished by DOX/sh-1@PLT treatment (Fig. 6B). On the 9th, 18th, and 28th days, the DOX/sh-1@PLT-treated group had the lowest fluorescence intensity, and the mice in this group had the smallest tumor volume and weight, as well as the fewest lesions (Fig. 6C). In addition, we investigated how DOX/sh-1@PLT affected HCC growth in orthotopic xenograft HCC models. Silencing of circ_0000098 significantly reduced the growth of HCC in the livers of mice and made tumors more sensitive to DOX (Fig. 6D-F). These findings suggested that DOX/sh-1@PLT could effectively prevent the development of HCC and reduce tumor resistance to DOX *in vivo*.

### The circ_0000098/miR-383/MCUR1 signaling modulates HCC development *in vivo*

**Fig. 7:**
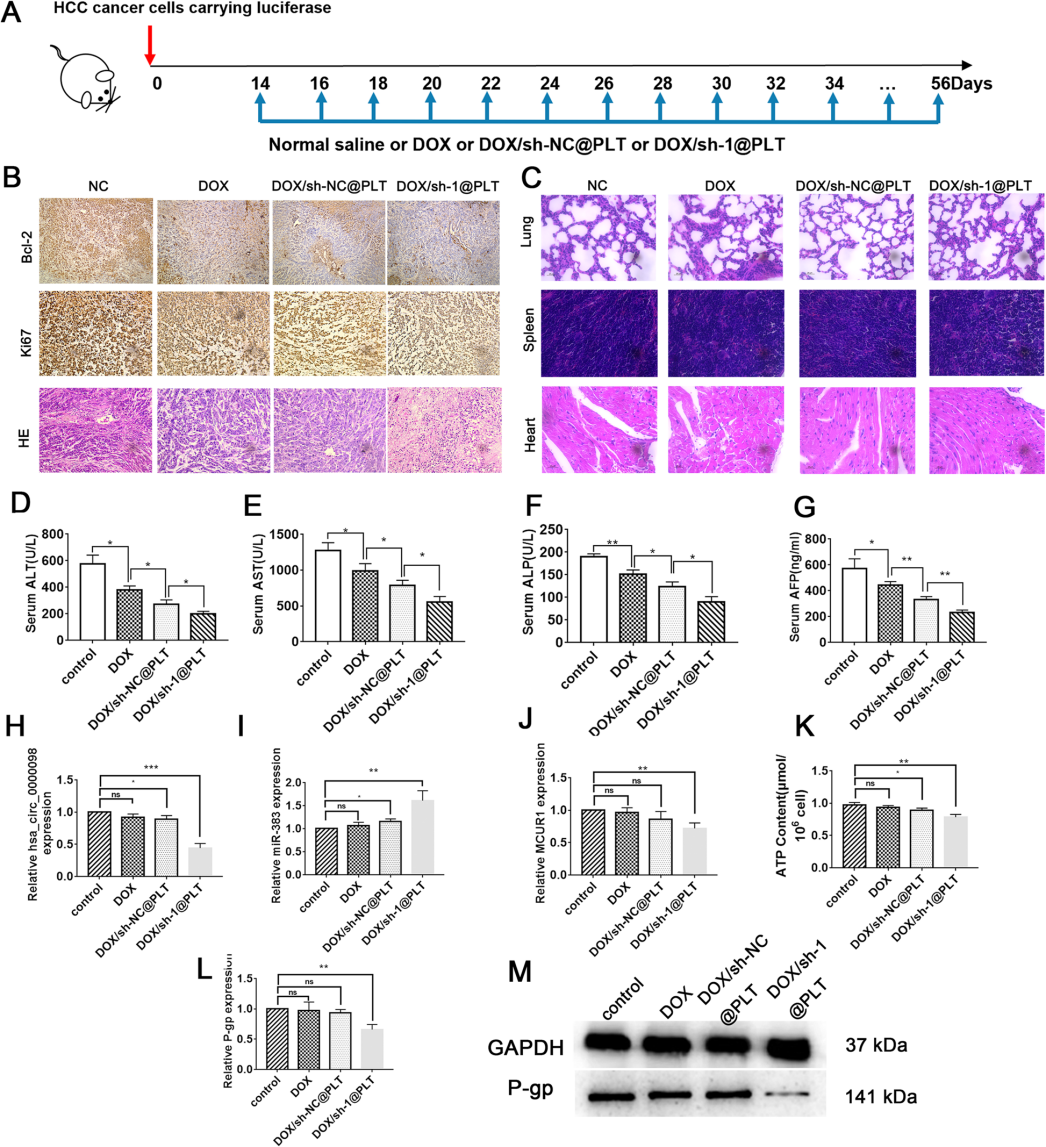
The circ_0000098/miR-383/MCUR1 signaling modulates HCC development *in vivo*. A Schematic illustration of the treatment regimen. **B** Immunohistochemical images and HE staining images of tumors. **C** HE staining images of lungs, spleens, and hearts. **D**-**G** ALT (**D**), AST (**E**), ALP (**F**), and AFP (**G**) levels were measured in the indicated groups. **H-J** RT-qPCR assays were used to detect circ_0000098 (**H**), miR-383 (**I**), and *MCUR1* (**J**) expression in the indicated groups. **K-L** Detection of ATP levels (**K**) and *P-gp* expression (**L**) in the indicated groups. **M** Western blotting analysis of P-gp expression in the indicated groups. *P < 0.05, **P < 0.01

**Fig. 8:**
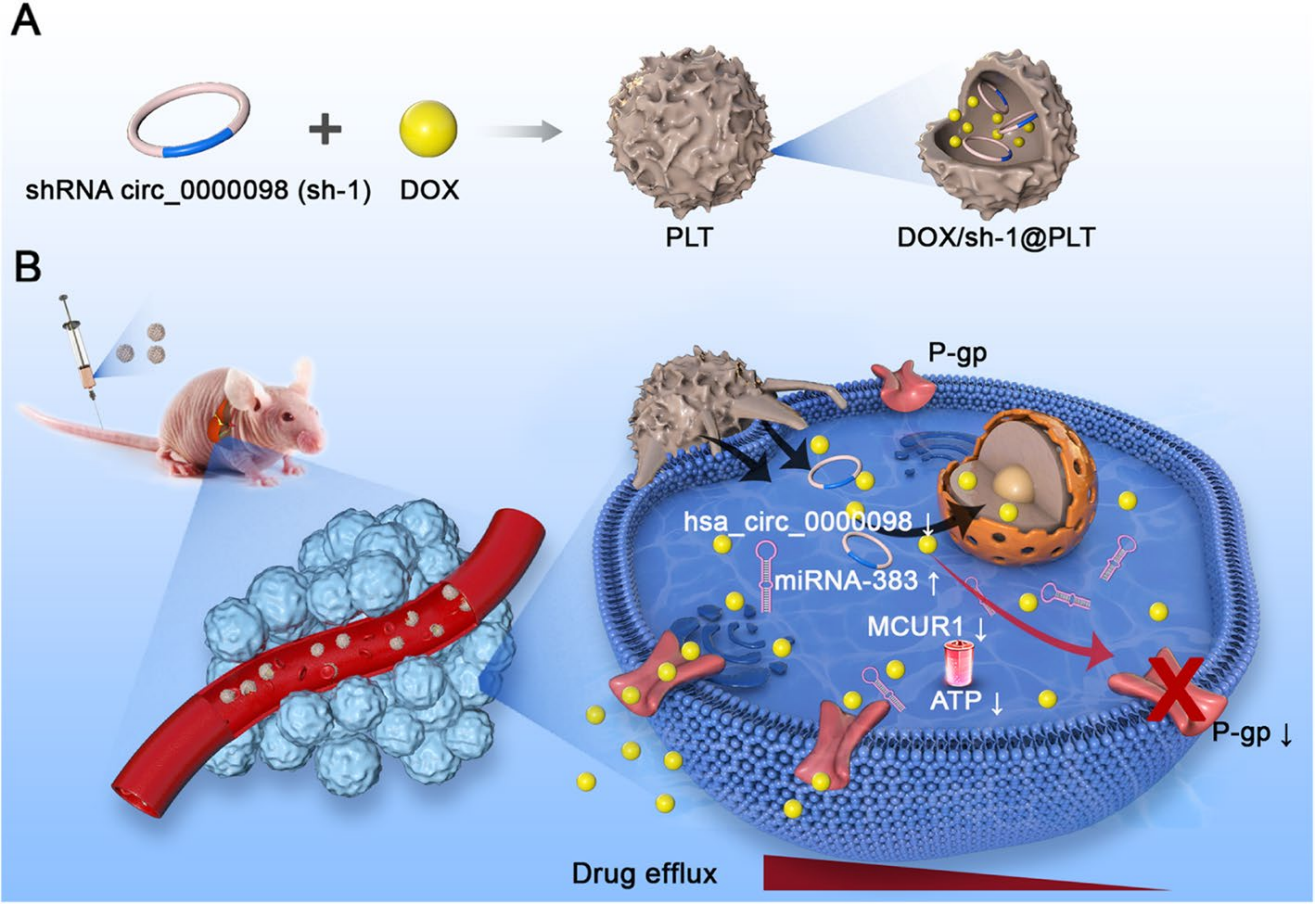
Schematic illustration of the role and mechanism of circ_0000098 in HCC. Circ_0000098 promotes HCC development through the miR-383/MCUR1 axis. Targeting circ_0000098 with DOX/sh-1@PLT may represent a feasible and practicable method for reducing DOX resistance in HCC

To investigate the involvement of the circ_0000098/miR-383/MCUR1 pathway during HCC development *in vivo*, physiological saline (NC), DOX, DOX/sh-NC@PLT, and DOX/sh-1@PLT were administered into HCC model mice from day 14 to day 56 (Fig. 7A). As expected, the DOX group and DOX/sh-NC@PLT group had smaller tumors compared to the NC group, however, the DOX/sh-1@PLT group had a tumor size reduction that tumors were difficult to detect (Fig. S8A). *In vivo* imaging technology was used to assess fluorescence intensity in nude mice, and we confirmed that those mice in the DOX/sh-1@PLT group had the lowest fluorescence intensity and the smallest tumor volume in the liver (Fig. S8B-C). Following that, ultraviolet spectrophotometry was performed to determine the presence of DOX in the tumor homogenate liquid, and we found that DOX/sh-1@PLT group had the greatest DOX concentration, suggesting that DOX/sh-1@PLT successfully inhibited DOX efflux (Fig. S8D). The immunohistochemical analysis further showed that tumor proliferation was decreased, while cell apoptosis was greatly increased in the DOX/sh-1@PLT group as compared to DOX and DOX/sh-NC@PLT group (Fig. 7B). HE staining subsequently indicated that the lungs, spleens, and heart tissues of the mice in the DOX/sh-NC@PLT group were in a normal condition, with no evident necrosis (Fig. 7C). More importantly, blood examination of the mice revealed that the levels of ALT, AST, ALP, and AFP dropped in the DOX/sh-1@PLT group compared to other groups, indicating that DOX/sh-1@PLT had a strong therapeutic effect on HCC (Fig. 7D-G). Finally, RT-qPCR experiments revealed that after DOX/sh-1@PLT treatment, the expression of circ_0000098 and *MCUR1* in tumor tissues was decreased, whereas miR-383 expression was induced in tumor tissues (Fig. 7H-J). We further found that downregulation of circ_0000098 could reduce the level of ATP and the expression of P-gp in tumor tissues of tumor-bearing mice (Fig. 7K-M). Together, our results supported that circ_0000098 facilitates HCC progression and promotes DOX resistance through the miR-383/MCUR1 axis (Fig. 8).

## Discussion

HCC has a significant global prevalence and is currently recognized as the third leading cause of cancer-related death, although its underlying mechanisms remain poorly understood. The majority of HCC patients are detected after the disease has advanced to the stage when surgical therapy is no longer viable. Therefore, chemotherapy has become one of the most commonly used treatments for patients with advanced HCC [24]. However, HCC is a highly drug-resistant malignancy, and its capacity to resist therapy significantly affects the effectiveness of anti-cancer drugs. Therefore, reducing HCC cell chemoresistance has become a critical priority. Many studies have shown that non-coding RNAs are intrinsically connected to tumor treatment resistance, with circRNAs standing out owing to their structural stability, specificity, and lengthy half-lives. CircRNAs may be used as useful therapeutic targets since they have a role in the development of drug resistance in many cancers. For example, Li et al. discovered that circARNT2 might control cisplatin resistance through the miR-155-5p/PDK1 pathway and has therapeutic potential for overcoming cisplatin resistance in HCC [25]. CircRNA_101505 enhances HCC sensitivity to cisplatin by sponging miR-103 [26]. CircPVT1 promotes cisplatin and methotrexate resistance through the miR-145-5p/ABCC1 axis [27]. In this work, a novel circRNA, circ_0000098, was discovered to be significantly elevated in HCC tissues and cell lines and might be used to efficiently identify HCC patients. Increased expression of circ_0000098 was associated with a worse HCC patient survival rate. According to *in vitro* cellular studies, knocking down circ_0000098 attenuated HCC cell proliferation and invasiveness, while generating cycle arrest and apoptosis in HCC cells. Also, silencing of circ_0000098 could impede the progression of HCC *in vivo*. To the best of our knowledge, for the first time, this study showed that circ_0000098 exhibits the oncogenic roles in hastening the malignant progression of HCC.

It is known that the majority of cytoplasmic circRNAs behave as miRNA sponges in tumor cells [15–17]. Using nucleoplasm separation studies, circ_0000098 was mostly detected in the cytoplasm of HCC cells. Using bioinformatics analysis, RT-qPCR, RIP, and dual-luciferase reporter assays, the existence of the circ_0000098/miR-383/MCUR1 axis in HCC cells was confirmed. In HCC tissues, circ_0000098 expression was negatively connected with miR-383 expression and positively correlated with *MCUR1* expression, according to our correlation analyses. We further demonstrated that circ_0000098 regulated MCUR1 expression by competitive bonding with miR-383. According to previous reports, miR-383 is dysregulated in several cancers and controls their growth [28, 29]. Long non-coding RNA CASC9-1 promotes cervical cancer progression by targeting miR-383 to upregulate MAPKAP1 [28]. CircCRIM1 also promotes the tumorigenesis of ovarian cancer by targeting the miR-383/ZEB2 axis [29]. In our study, *in vitro* experiments supported that circ_0000098 increases HCC development through the miR-383/MCUR1 axis, however, the detailed impact of this signaling on HCC remains to be further investigated.

MCUR1 is an important membrane protein for MCU-dependent mitochondrial calcium uptake [9–11]. MCUR1 binds to MCU and regulates calcium absorption in ruthenium red-sensitive cells in an MCU-dependent manner [9–11]. MCUR1 knockdown has little effect on MCU localization but abolishes Ca2^+^ absorption by activated mitochondria in permeabilized cells [9–11]. Depletion of MCUR1 inhibits oxidative phosphorylation and reduces cellular ATP [10–12]. Reducing drug efflux pumps (such as P-gp) and enhancing chemotherapeutic effectiveness have become crucial for the treatment of human malignancies. In our study, we discovered that circ_0000098 elevated ATP generation and the expression of the drug efflux pump P-gp in HCC cells possibly through the miR-383/MCUR1 axis. More research is required to fully understand the potential therapeutic advantages of circ_0000098 inhibitor on HCC. It has been reported that PLT-encapsulated sorafenib successfully increased HCC tissue necrosis, suggesting that PLT-encapsulated pharmaceuticals might be a new therapeutic option for HCC [30]. In our investigation, PLT was used as a drug carrier and it effectively encapsulated DOX and sh-1 to create DOX/sh-1@PLT. Our results suggested that DOX/sh-1@PLT accumulated higher in HCC cells and less in normal cells, with little impact on the lungs, spleens, and hearts of mice. More importantly, in the moue HCC models, DOX/sh-1@PLT efficiently suppressed the efflux of DOX inside the tumors of nude mice, dramatically decreased tumor volume, and showed favorable therapeutic effects. Therefore, circ_0000098 may serve as a potential therapeutic target for patients with advanced HCC.

## Conclusion

The present study demonstrated that circ_0000098 is highly expressed in HCC and supports its progression through the miR-383/MCUR1 axis. Inhibition of circ_0000098 with DOX/sh-1@PLT reduces P-gp expression, intracellular ATP levels, and DOX resistance. Our findings highlighted the crucial functions of circ_0000098 in HCC and suggested that circ_0000098 silencing in combination with DOX might be a feasible and practicable therapeutic strategy for HCC.

## Supplementary Information

**Additional file 1: Figure S1.** Cell apoptosis was detected with flow cytometry in HCC cells with circ_0000098 knockdown or overexpression. *P < 0.05, **P < 0.01, ***P < 0.001. **Figure S2.** Silencing of circ_0000098 repressed HCC tumorigenesis* in vivo*. (A) Images of nude mice on days 7, 14, and 21 after subcutaneous inoculation with the indicated HCC cells. (B-D) The growth curve (B), tumor volume (C), and weight (D) of transplanted tumors were measured in the indicated groups. (E) Expression of hsa_circ_0000098 in the indicated groups. (F) HE-stained images and Ki67 expression images of tumors in the indicated groups. *P < 0.05. **Figure S3.** The expression of miR-383 and *MCUR1* in HCC cells. (A) The expression of miR-383 in TCGA liver cancer tissues and normal tissues (ENCORI database). (B) The relationship between the expression of miR-383 and the survival of HCC patients (KM plotter database). (C, D) Detection of miR-383 expression in BEL-7404 (C) and HCC-LM3 (D) cells transfected with control or miR-383 mimics (E) The expression of* MCUR1* in TCGA liver cancer tissues and normal tissues (ENCORI database) (F) The relationship between the expression of *MCUR1 *and the survival of HCC patients (KM plotter database). (G, H) Detection of *MCUR1* expression in BEL-7404 (G) and HCC-LM3 (H) cells transfected with control shRNA (sh-NC) or MCUR1 shRNA (sh-MCUR1). **P < 0.01, ***P < 0.001. **Figure S4.** KEGG pathway enrichment analysis (A) and GO biological processes annotation (B) of predicted miR-383 targets (ENCORI database). **Figure S5.** Effects of circ_0000098 and MCUR1 expression on HCC cell proliferation. (A-B) CCK-8 assays were performed in HCC-LM3 (A) and BEL-7404 (B) cells transfected as indicated. *P < 0.05, **P < 0.01, ***P < 0.001. **Figure S6.** The expression of circ_0000098, miR-383, and MCUR1 in HCC tissues. (A-C) RT-qPCR assays were performed to detect circ_0000098 (A), miR-383 (B) and *MCUR1* (C) expression in sh-1 or sh-NC-transfected groups (D) The relationship between the expression of *ABCB1 *(P-gp) and the survival of HCC patients (KM plotter database). *P < 0.05, **P < 0.01. **Figure S7.** Detection of the excitation process of DOX/sh-1@PLT by particle size analysis technology. **Figure S8.** DOX/sh-1@PLT inhibited HCC progression* in vivo*. (A) Detection of tumor size using computed tomography. (B) Detection of fluorescence intensity alterations in mouse HCC models. (C) Images of tumors from mouse HCC models. (D) Detection of DOX accumulation in the indicated tumors. NS: not significant. *P<0.05.

## Data Availability

All of the data and material in this paper are available when requested. The public datasets analyzed during the current study are available in the repositories listed below: • Gene Expression Omnibus: GSE155949 https://www.ncbi.nlm.nih.gov/geo/query/acc.cgi?acc=GSE155949 • Gene Expression Omnibus: GSE101850 https://www.ncbi.nlm.nih.gov/geo/query/acc.cgi?acc=GSE101850

## Abbreviations

HCC: Hepatocellular carcinoma
MDR: Multiple drug resistance
DOX: Doxorubicin
MCUR1: Mitochondrial calcium uniporter regulatory factor 1
CircRNA: Circular RNA
miRNA: MicroRNA
PLT: Platelet
P-gp: P-glycoprotein
ABC: ATP-binding cassette
