# Supplementary material for: Hsa_circ_0000098 is a novel therapeutic target that promotes hepatocellular carcinoma development and resistance to doxorubicin
Source: J Exp Clin Cancer Res. 2022 Sep 7;41:267. doi: 10.1186/s13046-022-02482-3 (PMC9450443; doi:10.1186/s13046-022-02482-3)
Supplement: Supplementary file 1 — Additional file 1: Figure S1. Cell apoptosis was detected with flow cytometry in HCC cells with circ_0000098 knockdown or overexpression. *P < 0.05, **P < 0.01, ***P < 0.001. Figure S2. Silencing of circ_0000098 repressed HCC tumorigenesis in vivo. (A) Images of nude mice on days 7, 14, and 21 after subcutaneous inoculation with the indicated HCC cells. (B-D) The growth curve (B), tumor volume (C), and weight (D) of transplanted tumors were measured in the indicated groups. (E) Expression of hsa_circ_0000098 in the indicated groups. (F) HE-stained images and Ki67 expression images of tumors in the indicated groups. *P < 0.05. Figure S3. The expression of miR-383 and MCUR1 in HCC cells. (A) The expression of miR-383 in TCGA liver cancer tissues and normal tissues (ENCORI database). (B) The relationship between the expression of miR-383 and the survival of HCC patients (KM plotter database). (C, D) Detection of miR-383 expression in BEL-7404 (C) and HCC-LM3 (D) cells transfected with control or miR-383 mimics (E) The expression of MCUR1 in TCGA liver cancer tissues and normal tissues (ENCORI database) (F) The relationship between the expression of MCUR1 and the survival of HCC patients (KM plotter database). (G, H) Detection of MCUR1 expression in BEL-7404 (G) and HCC-LM3 (H) cells transfected with control shRNA (sh-NC) or MCUR1 shRNA (sh-MCUR1). **P < 0.01, ***P < 0.001. Figure S4. KEGG pathway enrichment analysis (A) and GO biological processes annotation (B) of predicted miR-383 targets (ENCORI database). Figure S5. Effects of circ_0000098 and MCUR1 expression on HCC cell proliferation. (A-B) CCK-8 assays were performed in HCC-LM3 (A) and BEL-7404 (B) cells transfected as indicated. *P < 0.05, **P < 0.01, ***P < 0.001. Figure S6. The expression of circ_0000098, miR-383, and MCUR1 in HCC tissues. (A-C) RT-qPCR assays were performed to detect circ_0000098 (A), miR-383 (B) and MCUR1 (C) expression in sh-1 or sh-NC-transfected groups (D) The relationship between the ex [file 13046_2022_2482_MOESM1_ESM.pdf]

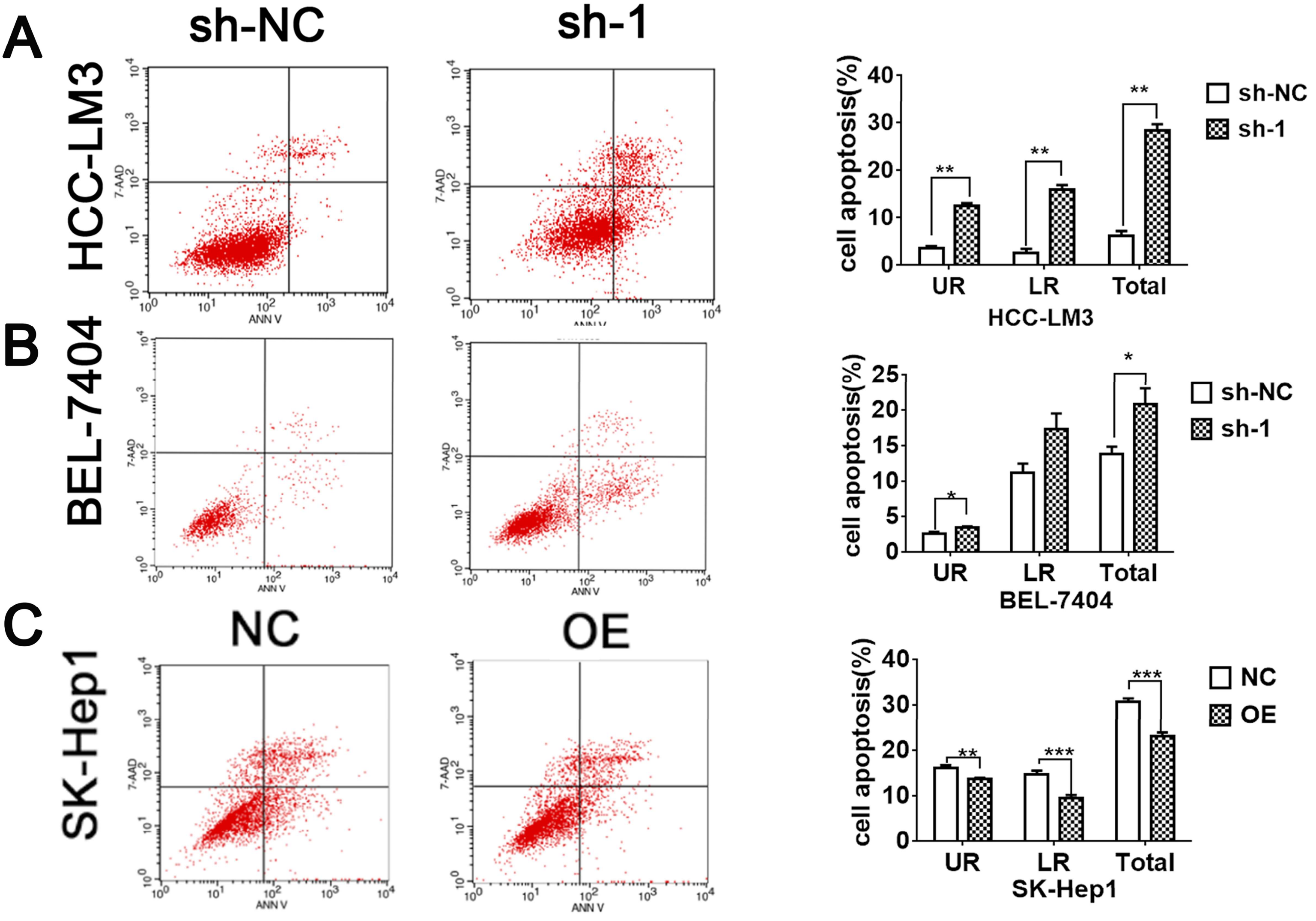
A
sh-NC
sh-1
HCC-LM3
B
BEL-7404
C
NC
OE
SK-Hep1
7-AAD
ANN V
cell apoptosis(%)
UR
LR
Total
**
*
***

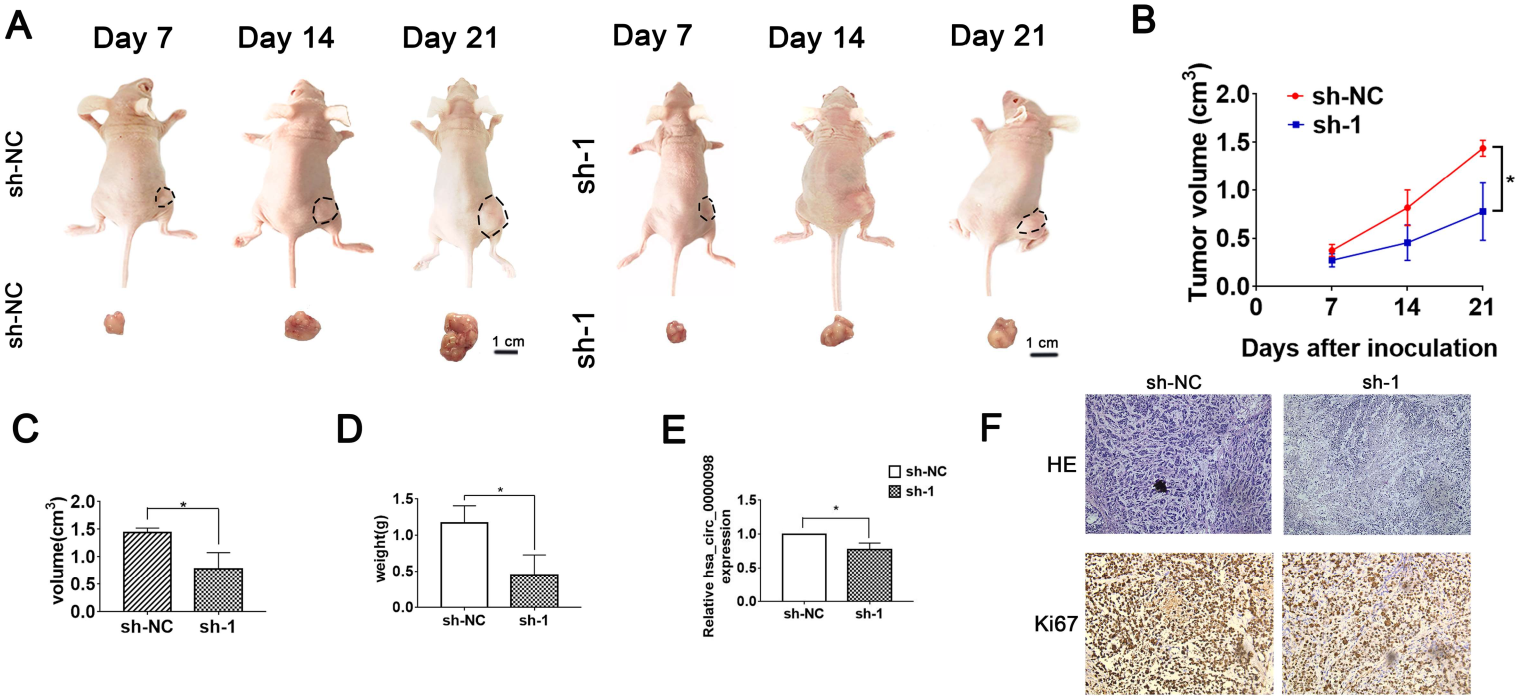
A
Day 7
Day 14
Day 21
Day 7
Day 14
Day 21
sh-NC
sh-NC
sh-1
sh-1
1 cm
1 cm
B
Tumor volume (cm3)
sh-NC
sh-1
Days after inoculation
C
volume(cm3)
sh-NC
sh-1
D
weight(g)
sh-NC
sh-1
E
Relative hsa_circ_0000098 expression
sh-NC
sh-1
F
sh-NC
sh-1
HE
Ki67

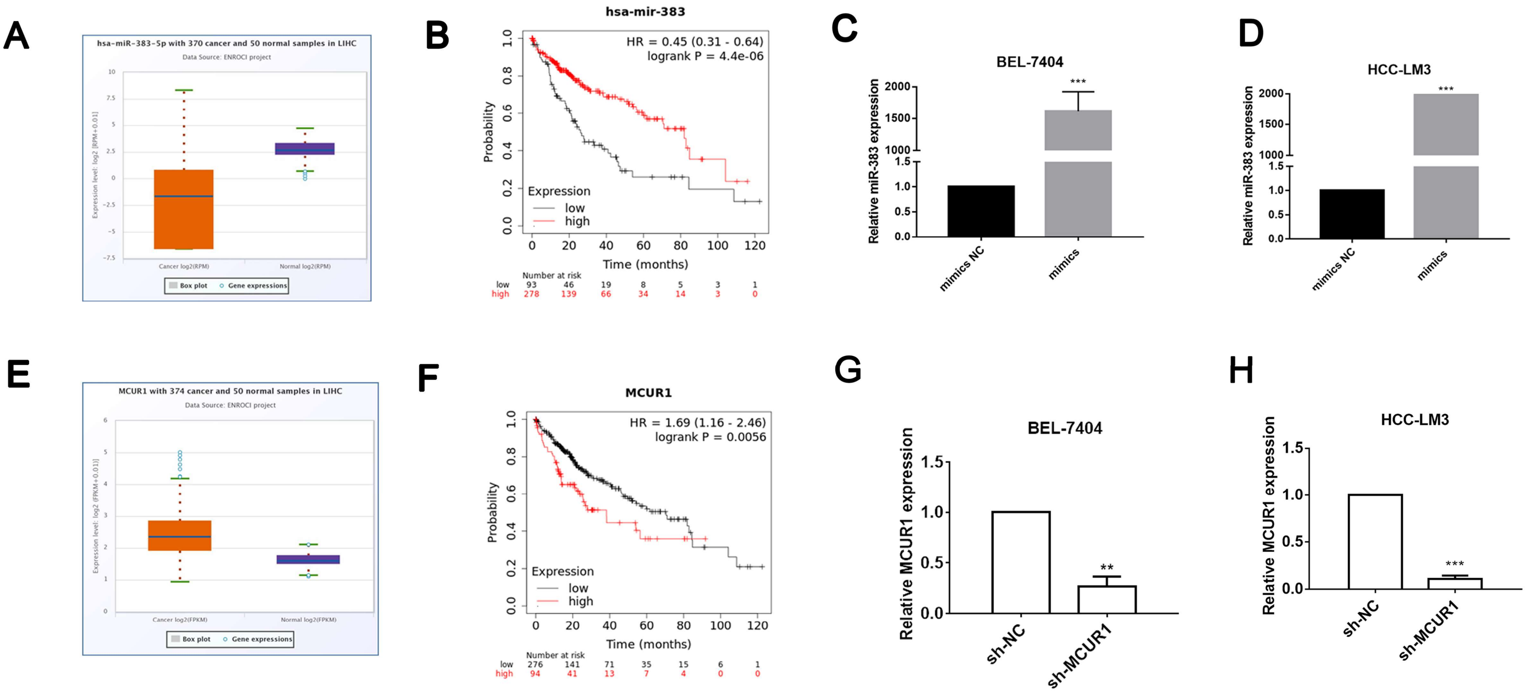
A
hsa-miR-383-5p with 370 cancer and 50 normal samples in LIHC
Box plot
Gene expressions
B
hsa-mir-383
HR = 0.45 (0.31 - 0.64)
logrank P = 4.4e-06
Probability
Expression
low
high
Time (months)
Number at risk
low 93 46 19 8 5 3 1
high 278 139 66 34 14 3 0
C
BEL-7404
Relative miR-383 expression
***
mimics NC
mimics
D
HCC-LM3
Relative miR-383 expression
***
mimics NC
mimics
E
MCUR1 with 374 cancer and 50 normal samples in LIHC
Box plot
Gene expressions
F
MCUR1
HR = 1.69 (1.16 - 2.46)
logrank P = 0.0056
Probability
Expression
low
high
Time (months)
Number at risk
low 276 141 71 35 15 6 1
high 94 41 13 7 4 0 0
G
BEL-7404
Relative MCUR1 expression
**
sh-NC
sh-MCUR1
H
HCC-LM3
Relative MCUR1 expression
***
sh-NC
sh-MCUR1

A

pathwayName	log10(pval)
KEGG_Pathways_In_Cancer	-5.55579
KEGG_Regulation_Of_Actin_Cytoskeleton	-3.55793
KEGG_Renal_Cell_Carcinoma	-3.61839
KEGG_P53_Signaling_Pathway	-3.69142
KEGG_Focal_Adhesion	-3.78243
KEGG_Vasopressin_Regulated_Water_Reabsorption	-3.40194
KEGG_Fc_Gamma_R_Mediated_Phagocytosis	-3.86731
KEGG_Pancreatic_Cancer	-3.09634
KEGG_Chronic_Myeloid_Leukemia	-2.90887
KEGG_Glioma	-2.91483
KEGG_Endocytosis	-2.73851
KEGG_Gnrh_Signaling_Pathway	-2.74670
KEGG_Neurotrophin_Signaling_Pathway	-2.43671

B

pathwayName	log10(pval)
GO_Activation_Of_Gtpase_Activity	-3.27701
GO_Adherens_Junction_Assembly	-3.07185
GO_Alpha_Beta_T_Cell_Differentiation	-2.71895
GO_Anatomical_Structure_Formation_Involved_In_Morphogenesis	-3.46456
GO_Camp_Mediated_Signaling	-2.78799
GO_Cell_Cycle	-4.45041
GO_Cell_Cycle_Checkpoint	-4.07137
GO_Cell_Cycle_Process	-3.26651
GO_Cell_Death	-3.12568

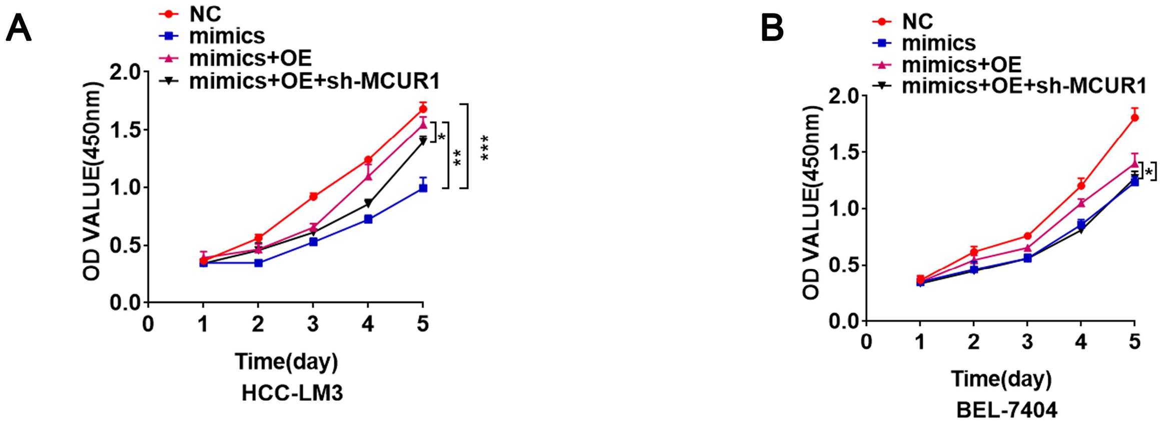
A
NC
mimics
mimics+OE
mimics+OE+sh-MCUR1
OD VALUE(450nm)
2.0
1.5
1.0
0.5
0.0
0
1
2
3
4
5
*
**
***
Time(day)
HCC-LM3
B
NC
mimics
mimics+OE
mimics+OE+sh-MCUR1
OD VALUE(450nm)
2.0
1.5
1.0
0.5
0.0
0
1
2
3
4
5
*
**
***
Time(day)
BEL-7404

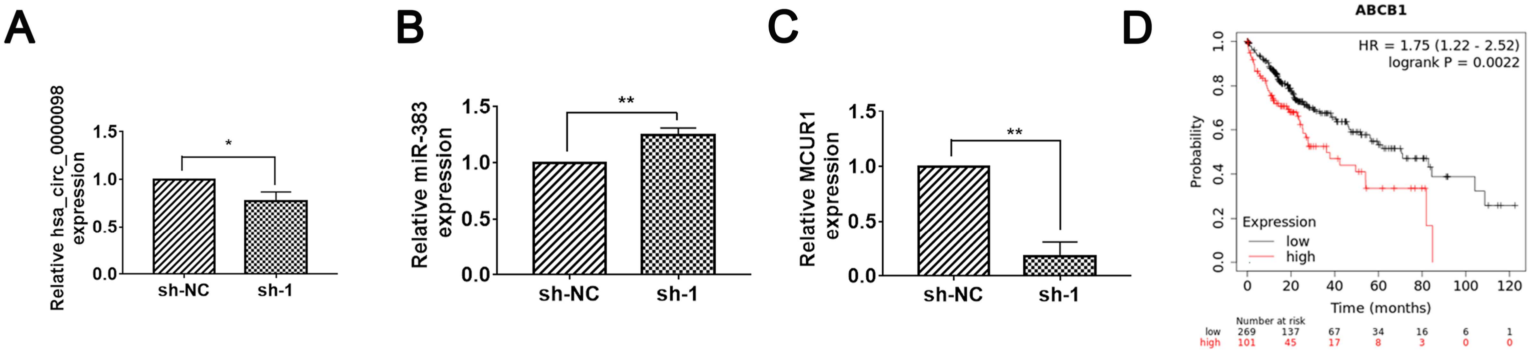
A
Relative hsa_circ_0000098 expression
1.5
1.0
0.5
0.0
*
sh-NC
sh-1
B
Relative miR-383 expression
1.5
1.0
0.5
0.0
**
sh-NC
sh-1
C
Relative MCUR1 expression
1.5
1.0
0.5
0.0
**
sh-NC
sh-1
D
ABCB1
HR = 1.75 (1.22 - 2.52)
logrank P = 0.0022
Probability
1.0
0.8
0.6
0.4
0.2
0.0
Expression
low
high
0
20
40
60
80
100
120
Time (months)
Number at risk
low 269 137 67 34 16 6 1
high 101 45 17 8 3 0 0

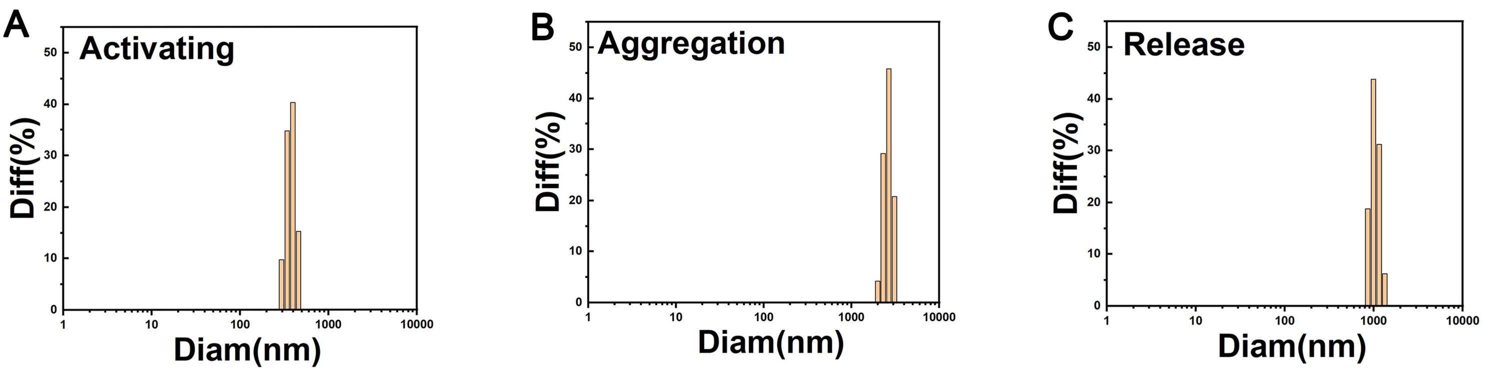
A
Activating
Diff(%)
Diam(nm)
B
Aggregation
Diff(%)
Diam(nm)
C
Release
Diff(%)
Diam(nm)

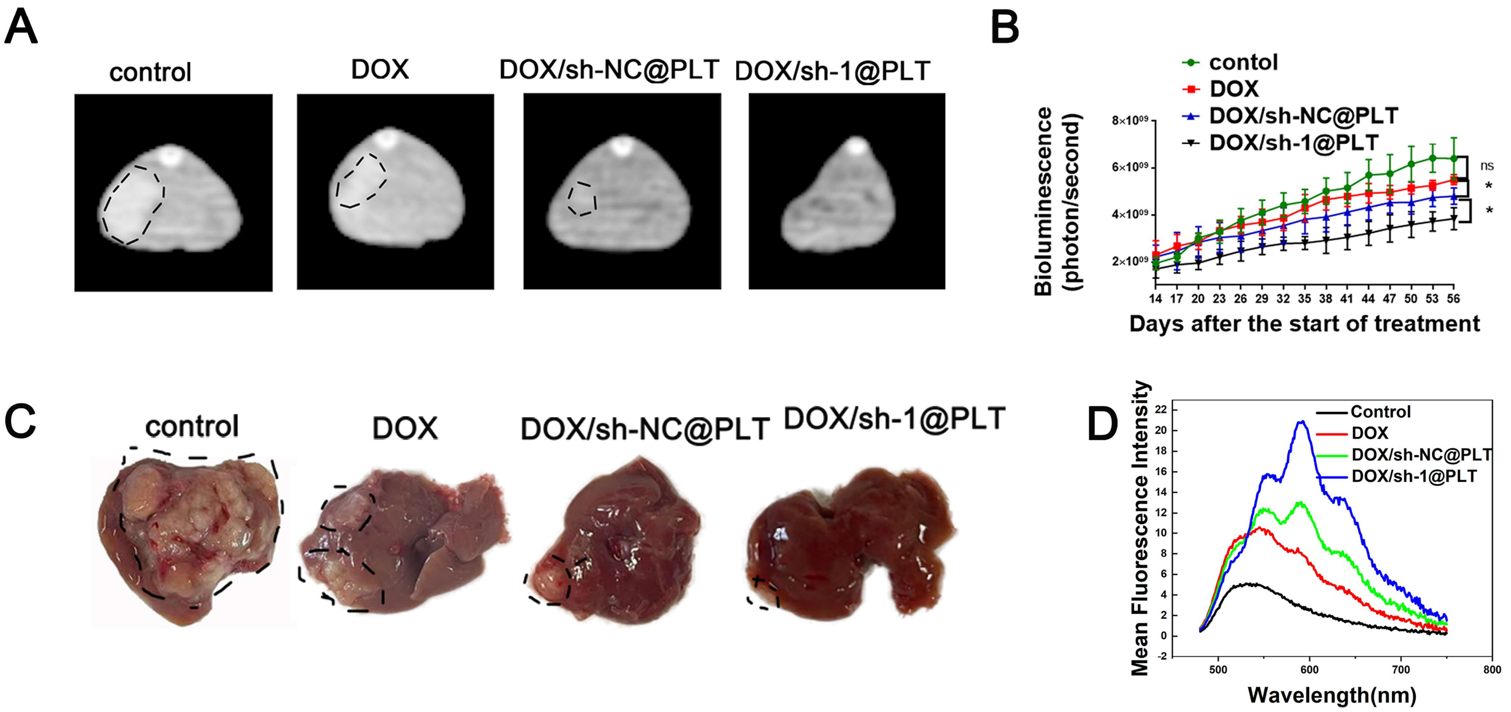
A
control
DOX
DOX/sh-NC@PLT
DOX/sh-1@PLT
B
contol
DOX
DOX/sh-NC@PLT
DOX/sh-1@PLT
Bioluminescence (photon/second)
Days after the start of treatment
ns
C
control
DOX
DOX/sh-NC@PLT
DOX/sh-1@PLT
D
Control
DOX
DOX/sh-NC@PLT
DOX/sh-1@PLT
Mean Fluorescence Intensity
Wavelength(nm)
